# Complete Genomic Sequence of Maize Rough Dwarf Virus, a Fijivirus Transmitted by the Small Brown Planthopper

**DOI:** 10.1128/genomeA.01529-15

**Published:** 2016-02-04

**Authors:** Mingfang Lv, Li Xie, Jian Yang, Jianping Chen, Heng-Mu Zhang

**Affiliations:** State Key Laboratory Breeding Base for Zhejiang Sustainable Pest and Disease Control, Key Laboratory of Plant Protection and Biotechnology, Ministry of Agriculture, Zhejiang Provincial Key Laboratory of Plant Virology, Institute of Virology and Biotechnology, Zhejiang Academy of Agricultural Sciences, Hangzhou, China

## Abstract

The nucleotide sequences of the 10 genomic segments of an Italian isolate of maize rough dwarf virus (MRDV) were determined. This first complete genomic sequence of MRDV will help understand the phylogenetic relationships among group 2 fijiviruses and especially the closely related rice black-streaked dwarf virus, which is also found to naturally infect maize.

## GENOME ANNOUNCEMENT

Maize rough dwarf virus (MRDV) was first described in Italy in the late 1940s (1) and is one of four recognized species in group 2 of the genus *Fijivirus*, family *Reoviridae* (2). MRDV naturally infects maize and causes a severe and economically important disease primarily in Europe and the Middle East (1, 3). Infected maize plants are typically stunted, with swellings along the veins on the undersides of leaves and on the sheaths of leaves and ears, and they are far less productive compared to healthy maize plants. MRDV shares many features with rice black-streaked dwarf virus (RBSDV), another group 2 fijivirus. Both viruses can be transmitted to maize by the planthopper *Laodelphax striatellus* in a persistent manner and induce similar severe growth abnormalities (4). They have a close serological relationship, but there are important biological differences. RBSDV naturally infects rice, but MRDV does not, and while MRDV is transmitted through the eggs of the vector insect, RBSDV is not (4).

The nucleotide sequences of each of the 10 genomic RNAs of RBSDV have been determined (5, 6) but sequence information from MRDV is more limited (7). We have therefore determined the complete genomic sequence of an Italian isolate of MRDV to improve our understanding of the relationship between the two viruses and to assist in studies to correlate genome structure and function. Viral double-stranded RNA purification, reverse transcription-polymerase chain reaction (RT-PCR) amplification, cloning, and sequencing were performed, as previously described (6, 8). The inserts were sequenced using the BigDye Terminator version 3.1 cycle sequencing kit on an ABI PRISM 3730 DNA sequencer (PerkinElmer Applied Biosystems, USA). Sequences were assembled using the DNAman version 6.0 program (Lynnon Corporation, Canada).

The total 10-segment genome of MRDV was composed of 29,144 nucleotides (nt), which is 3 nt longer than the RBSDV genome and is similar in organization to RBSDV and the other group 2 fijiviruses that have been sequenced (Mal de Rio Cuarto virus [MRCV] and southern rice black-streaked dwarf virus [SRBSDV]). The extreme 5′ and 3′ ends of the sense strand of each segment had the sequence 5′-AAGTTTTTT… CAGCTA(G)T(C/A)T(C)GTC-3′, which is similar to the conserved terminal sequences reported from RBSDV, MRCV, and SRBSDV segments (7–9). A perfect or imperfect 6- to 10-bp inverted repeat was identified immediately adjacent to the conserved terminal sequences in each genomic segment. Each of the genome segments S1 to S4, S6, S8, and S10 encodes a single major protein, while S5, S7, and S9 each had two nonoverlapping or partially overlapping open reading frames (ORFs). The 13 ORFs share the highest homologies (81 to 97%) with those of the corresponding segments of RBSDV. S1 contained the major motifs characteristic of an RNA-dependent RNA polymerase (RdRp) (10), which can be aligned with those from the RdRp proteins of other reoviruses and which is thought to be a minor component of the core particle. RBSDV, MRDV, RBSDV-2, and MRCV were always closely clustered together in phylogenetic analyses of their segments, and the new sequences of MRDV will be important for determining the appropriate taxonomic positions of these viruses, which sometimes have been considered isolates of a single species (11).

### Nucleotide sequence accession numbers.

The complete genomic sequence of MRDV has been deposited in the GenBank/EMBL/DDBJ databases with the accession numbers HQ637550 to HQ637559 corresponding to genomic segments 1 to 10, respectively.
